# Pilot study of a new telehealth program for depressed older adults: The Positive Experience Project (P.E.P.)

**DOI:** 10.1093/geroni/igab046.2401

**Published:** 2021-12-17

**Authors:** Dolores Gallagher-Thompson, Nancy Morioka-Douglas, B J Fogg, Mei- Le Keck

**Affiliations:** 1 Stanford University School of Medicine, LOS ALTOS, California, United States; 2 Stanford University School of Medicine, Palo Alto, California, United States; 3 Stanford Medicine, Stanford, California, United States; 4 Stanford Health Care, Palo Alto, California, United States

## Abstract

Social isolation, logistical challenges, and limited access to mental health providers who accept Medicare contribute to older adults having a higher risk for untreated depression. Primary care providers (PCPs) are strained due to time demands and lack of training in behavioral activation and similar therapies. This study was designed to reduce depression in older adult primary care patients without burdening their PCPs. We evaluated whether outpatients age 65+ with mild to moderate self-reported depressive symptoms (measured by PHQ-8) benefited from a brief evidence-informed program, the Positive Experience Project(PEP). This is derived from the tenets and practices of both behavioral activation and the “tiny habits” program. The former emphasizes the value of increasing engagement in everyday positive activities to lift mood; the latter provides a method to enhance success by encouraging patients to link these activities with existing habitual behaviors, and to celebrate completion. A script was written for each of four 30-minute sessions conducted in small groups using a telehealth platform. An analysis of patients of the first author yielded 50 eligible patients. 8 were invited to participate and 7 did. The group’s mean PHQ-8 score pre-intervention was 10.1; post-intervention (4 weeks later), the average was 6.1 (P=.039). The use of “scripts” that guide the PCP through the visit enhances adoption. And PCPs can bill for these 30-minute sessions, making it feasible to help patients receive treatment for their depression. These promising results are currently being replicated by additional PCPs and their data will be included in the poster presentation.

